# BaTiO_3_ Nanoparticle Interfaces in Contact: Ferroelectricity Drives Tribochemically Induced Oxygen Radical Formation

**DOI:** 10.1021/acs.langmuir.4c03390

**Published:** 2024-12-13

**Authors:** Korbinian Aicher, Thomas Berger, Oliver Diwald

**Affiliations:** Department of Chemistry and Physics of Materials, Paris-Lodron University Salzburg, Jakob-Haringer-Straße 2a, A-5020 Salzburg, Austria

## Abstract

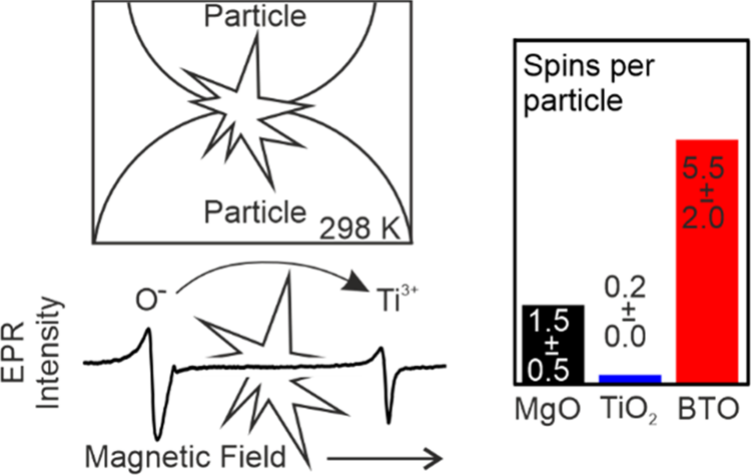

Chemical transformations at metal oxide interfaces that are triggered by mechanical energy set the basis for applications in the fields of tribo- and mechanochemistry, ceramic and composite processing, and piezoelectric devices. We investigated the early stages of tribochemically initiated radical chemistry of structurally well-defined TiO_2_ and BaTiO_3_ nanoparticles in argon or in oxygen atmosphere. Electron paramagnetic resonance spectroscopy enabled the determination of the chemical nature and concentration of paramagnetic surface species which form upon uniaxial powder compaction at room temperature. Trapped hole centers (O^–^) as well as trapped or scavenged electrons (Ti^3+^ or O_2_^–^, respectively) were analyzed as products of mechanical surface activation. For ferroelectric BaTiO_3_ nanoparticles, we found that the spontaneous polarization effects of the oxide lattice increase the yield of paramagnetic surface species by a factor >20 as compared to paraelectric TiO_2_ nanoparticles. Comparison with UV excitation experiments, where the energy required to drive the corresponding charge separation phenomena is *h*ν ≥ 3.2 eV, indicates that the paramagnetic species that originate from uniaxial powder compaction in the dark result from mechanically induced surface redox processes that are supported by local flexoelectric potential differences.

## Introduction

Understanding how the impact of mechanical energy at particle contact interfaces transforms molecules and materials chemically is important for a variety of expanding application fields, such as mechanochemistry, tribochemistry and -catalysis, or piezoelectric nanogenerators.^1−7^ Typically, a tribochemical process is very complex, since the absorption of mechanical energy inside solids can produce a variety of materials transformations that range from tribocharging, triboemission of energy or electrons, to plastic deformation and amorphization of crystal lattices. Defect accumulation can occur both at interfaces and in the bulk, and metastable intermediates that serve as educts of subsequent solid-state reactions can emerge upon interfacial shear and friction. The nature of the material, its crystallinity and granularity, type of bonding, the intensity and mode of the mechanical energy applied, all together determine the resulting chemistry.^4^ What must not be overlooked in this context is the key role of composition, morphology, and abundance of various types of interfaces that are moved toward each other.

With large surface-to-volume ratios, nanoparticles offer significantly increased contact areas for friction and wear. Despite its relevance for tribochemistry inside powders, there are only a few fundamental experimental studies that focus on friction-induced formation of radicals and other paramagnetic defects in nanoparticle powders or in colloidal dispersions.^8−11^ Motivated by the need to assess the potential health risks and pathogenicity of silica, work on related particle systems has addressed grinding of quartz in different chemical environments.^12^ As another example, there have been studies that focused on the tribochemical activity of metal oxides like TiO_2_ with respect to the degradation of pollutants and dyes.^13^

In this work, we investigated the early steps during the transformation of mechanical energy into chemical reactions at BaTiO_3_ and TiO_2_ nanoparticles. These early steps correspond to electronic excitations of metal oxide nanoparticle interface structures that are followed by the emission of energy and/or electrons, which for mechanistic studies need to be detected directly.^3,14−16^ For anhydrous MgO nanocubes we found that powder compaction with uniaxial pressures (*p* ≥ 5 MPa), as it can be achieved by gentle manual rubbing, already excites energetic electron–hole pairs and generates oxygen radicals at interfacial defect structures.^17,18^ Characteristic spectroscopic property changes (UV–vis and electron paramagnetic resonance, EPR) that are indicative of charge separation effects at the nanograin interfaces were measured. Photoexcitation of the same materials was performed as a reference experiment and yielded identical EPR and optical spectroscopic fingerprints, providing for the first time molecular-level information on the pressure-induced separation and interfacial transfer of tribo-emitted charges in nanoparticle ensembles.^17^ Theoretical calculations at a quantum level explored different local contact configurations that could facilitate charge separation and consecutive interfacial charge transfer, but additional driving forces such as the flexoelectric potential difference are needed to explain the experimental observations made.^17^

Related to piezocatalysis, it was recently shown that BaTiO_3_ nanoparticles can trigger mechanoredox polymerization reactions in the solid state while retaining small traces of adsorbed residual water on the particle surfaces.^19^ Tribochemically generated OH radicals, which cannot be directly detected as such but can be monitored by spin trapping, play an important role as initiator of a radical polymerization reaction. For a mechanistic understanding of the intergranular surface chemistry that follows mechanical grain activation a defined starting point with model particle systems and adsorbate-free grain surfaces is needed.^20,21^

Here we address the question, whether rubbing of nanoparticle powders of ferroelectric BaTiO_3_ and paraelectric TiO_2_ as wide band gap semiconductors in anhydrous atmospheres (Ar, O_2_) leads to precursor states for any type of subsequent radical-driven reaction in between the grains of the nanoparticle compact.^17,18^ Motivated by their use as photocatalysts, several electron paramagnetic resonance (EPR) spectroscopy studies have focused on light-induced charge separation on the different TiO_2_ polymorphs.^22−28^ Moreover, the ferroelectric properties of BaTiO_3_, which originate from the noncentrosymmetric structure of the perovskite lattice and give rise to spontaneous polarization, are expected to also enhance charge separation. Such promotional effects can be extremely useful for tuning the reactivity and activity of catalysts, photocatalysts, sensors, and nanogenerators.

For this comparative study, we combine nanoparticle synthesis in the gas phase with subsequent thermal processing in defined gas atmospheres to prepare TiO_2_ or BaTiO_3_ nanoparticle powders with comparable size distributions and clean and adsorbate-free particle surfaces (i.e., surfaces exempt from solvent molecules and synthesis-related organic adsorbates). We take advantage of the high structural and compositional definition of the nanoparticle powders and the activation of their surfaces by adsorbate removal that is based on pretreatment under dynamic high vacuum conditions. Potential pressure-induced charge separation effects will be rationalized in terms of their oxide-specific electronic structure, i.e., a comparison between paraelectric TiO_2_ and ferroelectric BaTiO_3_.

## Experimental Section

### Materials Synthesis

BaTiO_3_ nanoparticle powders were synthesized using a self-constructed flame spray pyrolysis (FSP) reactor that is described in more detail in ref (29). The synthesis and preparation of the precursor follow the work of Schädli et al.^30^ Barium acetate (1.01704.0500 pro analysis, EMSURE ACS) was dissolved in 2-ethylhexanoic acid (EHA) for 3 h at 120 °C under constant stirring and using a reflux condenser. Titanium tetraisopropoxide (TTIP, Sigma-Aldrich, 97%) was diluted in toluene (anhydrous, Sigma-Aldrich, 99.8%) under constant stirring at room temperature. The two precursor solutions having a molar ratio of 1:1 between titanium and barium and a volumetric ratio of 1 between toluene and EHA were then mixed and injected at a flow rate of 2 mL·min^–1^ by a syringe pump into the nozzle of the flame burner and atomized by the dispersion gas (O_2_, 3.0 l·min^–1^). The spray flame in the reactor system is ignited by a supporting flame, which is fueled by a mixture of methane (CH_4_, 1.5 l·min^–1^) and oxygen (O_2_, 2.0 l·min^–1^) and is surrounded by an oxygen sheath gas (O_2_, 5.0 l·min^–1^).

TiO_2_ nanoparticle powders were synthesized via metal–organic chemical vapor synthesis (MOCVS). The MOCVS reactor consists of a fused silica tube placed inside a cylindrical furnace and a preheating zone to evaporate the precursor (TTIP, Sigma-Aldrich, 97%) at *T* = 393 K, and argon gas transports the gaseous precursor from the preheating zone into the furnace, where decomposition occurs at 1073 K. Stable process conditions are guaranteed by the spatial separation of the precursor evaporation and the reaction zone. Continuous pumping keeps the residence time of the resulting nuclei within the reactor short and prevents substantial coarsening and coalescence.

### Materials Processing

#### Thermal Annealing

Powder annealing was performed at temperatures up to 973 K in dedicated fused silica cells attached to a high vacuum rack, which allows for pressures as low as p(O_2_) < 10^–5^ mbar and defined gas atmospheres. Sample heating up to 973 K is described by a stepwise temperature increase of 100 K with a heating rate of 10 K·min^–1^. The next heating step is initiated as the pressure drops below *p* < 1·10^–5^ mbar. At 973 K the sample was dwelled for 60 min in vacuum. Afterward, molecular oxygen [p(O_2_) = 650 mbar for BTO, p(O_2_) = 20 mbar for TiO_2_] was added for 30 min, followed by evacuation (*p* < 5·10^–6^ mbar) for 30 min. Molecular oxygen was added again and dwelled for another 30 min. Afterward, the system is evacuated (*p* < 5·10^–6^ mbar), and fresh molecular oxygen is added prior to sample cooling to 200 °C. Once the sample temperature drops below 200 °C, the sample cell is evacuated (*p* < 5·10^–6^ mbar) and kept under dynamic high vacuum conditions for further cooling.

#### Powder Compaction

Powder compaction was performed via cold uniaxial pressing resulting in a regular disk-shaped specimen. For this purpose, the powder was transferred into the cavity (*d* = 13 mm) of a compaction tool (FTIR Pellet Dies, Specac) and uniaxially compressed with a hydraulic press (Atlas manual hydraulic press 15T, Specac) applying a pressure of *p* = 74 MPa for 1 min to obtain green compacts. To minimize water adsorption and to utilize the electron affinity of O_2_, powder compaction was carried out in a glovebag filled with pure oxygen and at room temperature.

### Materials Characterization

#### X-ray Diffraction

Continuous scan powder X-ray diffraction (XRD) data were collected at room temperature in coupled Theta–Theta mode on a Bruker D8 Advance with DaVinci design diffractometer. The instrument has a goniometer radius of 280 mm and is equipped with a fast solid-state Lynxeye detector and an automatic sample changer. Powder samples were prepared on a single-crystal silicon zero-background sample holder. Using Cu Kα_1,2_ radiation (λ = 154 pm) data acquisition was performed between 5 and 80.5° 2Θ with a step size of 0.02° and opened divergence and antiscatter slits at 0.3 and 4°, respectively. Data handling and qualitative phase analysis were performed with the Bruker software DIFFRAC.EVA V2.1. Crystallite domain sizes were derived from the integral reflection width by applying the Scherrer equation^31^ to the main diffraction feature.

#### Transmission Electron Microscopy

Transmission electron microscopy (TEM) data were acquired by using a JEOL JEM-F200 transmission electron microscope operating at 200 kV equipped with a cold field emission electron source. TEM images to access morphological and structural information were recorded using a TVIPS F216 2k by 2k CMOS camera. Evaluation of images acquired during TEM analysis was performed with either ImageJ (V1.52a) or EM Measure software from TVIPS.

#### N_2_-Sorption

N_2_-sorption analysis was performed at 77 K and the specific surface area was calculated by applying the model of Brunauer–Emmett–Teller (BET). Prior to sorption measurements with an ASAP 2020 adsorption porosimeter from Micromeritics GmbH, each sample was degassed under a vacuum at 573 K for 3 h. The BET surface area (*S*_BET_) was evaluated using adsorption data in a relative pressure range *p*/*p*_0_ of 0.06–0.21.

#### Continuous Wave X-band Electron Paramagnetic Resonance

Electron Paramagnetic Resonance (EPR) measurements were performed on a Bruker EMXplus-10/12/P/L spectrometer equipped with an EMX^Plus^ standard cavity and using an NMR teslameter that allows for the accurate determination of resonant field values. TiO_2_ and BTO green compact fragments were placed inside a Suprasil quartz glass tube (*d*_o_ = 5 mm, d_i_ = 3 mm) and connected to a high vacuum line with base pressures as low as *p* < 10^–5^ mbar. The corresponding setup also allows for the addition of pure gas atmospheres and for sample exposure to UV light. Photoexcitation of the compact fragments (green body) with polychromatic light (P_light_ = 78 mW), at room temperature in pure oxygen atmosphere (p(O_2_) = 30 mbar) was carried out using a 300 W Xe-arc lamp equipped with a water filter to exclude IR contributions from the excitation spectrum. EPR spectra were recorded at 10 K under high vacuum conditions using a waveguide cryogen-free system (Oxford Instruments). Typical parameters for spectrum acquisition were a microwave power of 1 mW, at a field modulation frequency of 100 kHz with a modulation amplitude of 0.2 mT. Spin systems of axial and rhombic g-tensor symmetries were modeled by using Gaussian and/or Lorentzian line shapes with the Matlab-based computational package EasySpin.

## Results and Discussion

**Figure 1:**
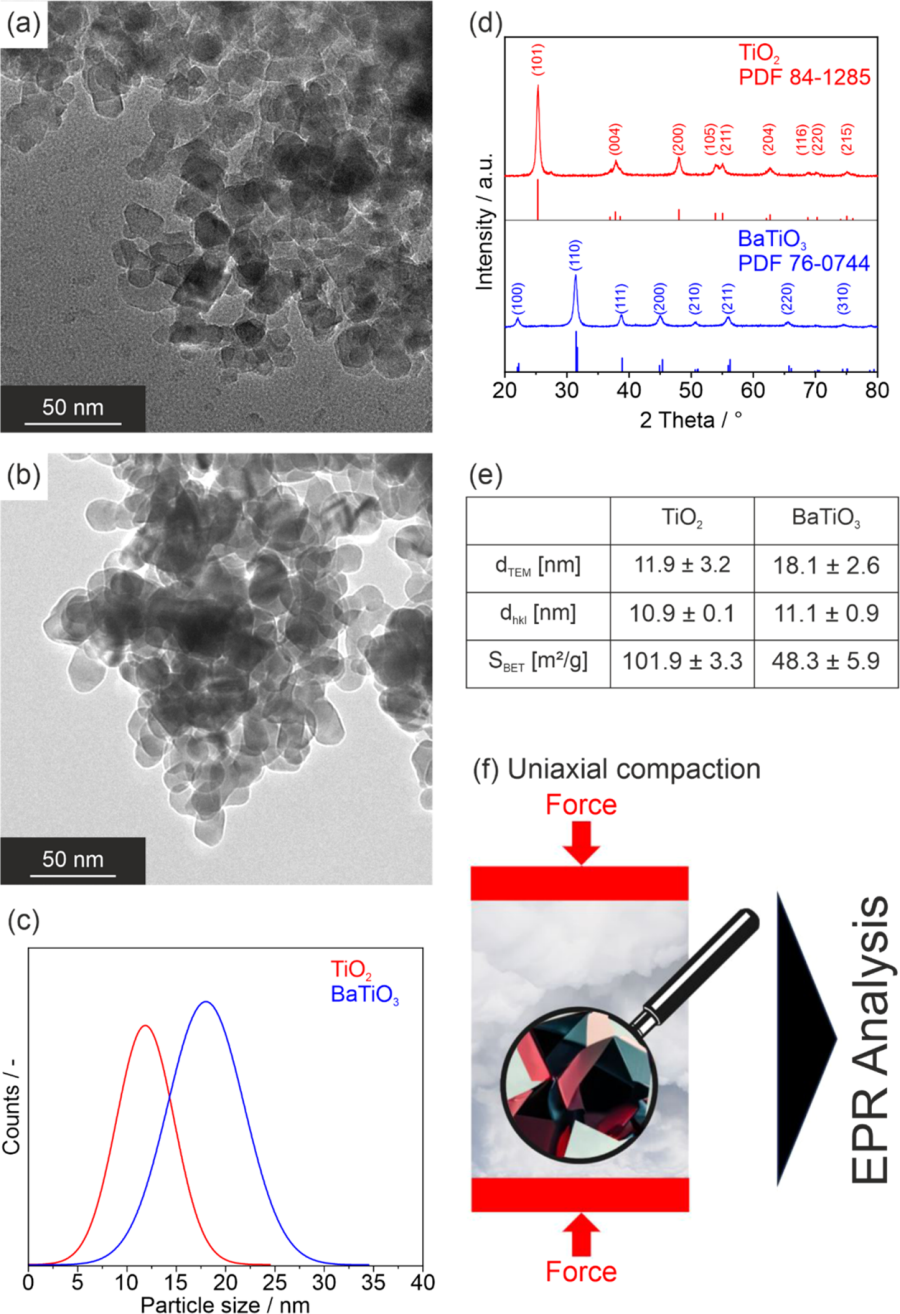
Transmission electron micrographs of (a) TiO_2_ and (b) BaTiO_3_ nanoparticle powders annealed at 973 K with the corresponding particle size distributions in panel (c). The powder X-ray diffraction patterns are shown in (d), and the values for median particle sizes, crystallite domain sizes, and BET surface areas are plotted in panel (e). (f) shows the methodological approach employed here for powder compaction in anhydrous Ar or O_2_ atmosphere and EPR analysis of the compacts carried out under dynamic high vacuum conditions.

The basic material properties of the two nanoparticle powders investigated are summarized in Figure 1. After thermal processing of the gas phase grown nanoparticle powders, both TiO_2_ (Figure 1a) and BaTiO_3_ (Figure 1b) nanoparticles are crystalline and do not show any type of specific grain faces. The particle size distributions determined by TEM data analysis are comparable (Figure 1c). While the median for the particle size distribution of TiO_2_ anatase nanocrystals agrees with the value for the crystallite domain size (Figure 1e), the mean crystallite domain size for BaTiO_3_, which deviates from the median, indicates deviations from the monocrystallinity of the individual particles. Amorphous areas were not detected during the high-resolution TEM measurements. Uniaxial powder compaction at room temperature (Figure 1f) was performed either in Ar or in the O_2_ atmosphere to yield disk-shaped compacts that were subsequently analyzed by electron paramagnetic resonance (EPR) spectroscopy under dynamic high vacuum conditions (*p* < 10^–5^ mbar).

Paramagnetic superoxide anions, i.e., O_2_^–^ states, and trapped hole centers, O^–^ states, provide the experimental basis for the here presented investigations. The values of individual g-parameters provide site-sensitive information about type and geometry of the interface sites. When the materials studied exhibit sufficiently high specific surface areas and a sufficiently high number of spectroscopically accessible surface sites, superoxide anions have been successfully used as surface probes^32−35^ to analyze different metal oxides in terms of their charge separation efficiency, as well as their catalytic and photocatalytic properties. Moreover, the quantitative analysis of the EPR signals also enables one to identify metal-oxide-specific trends in the charge separation properties of metal oxide particles.

**Figure 2:**
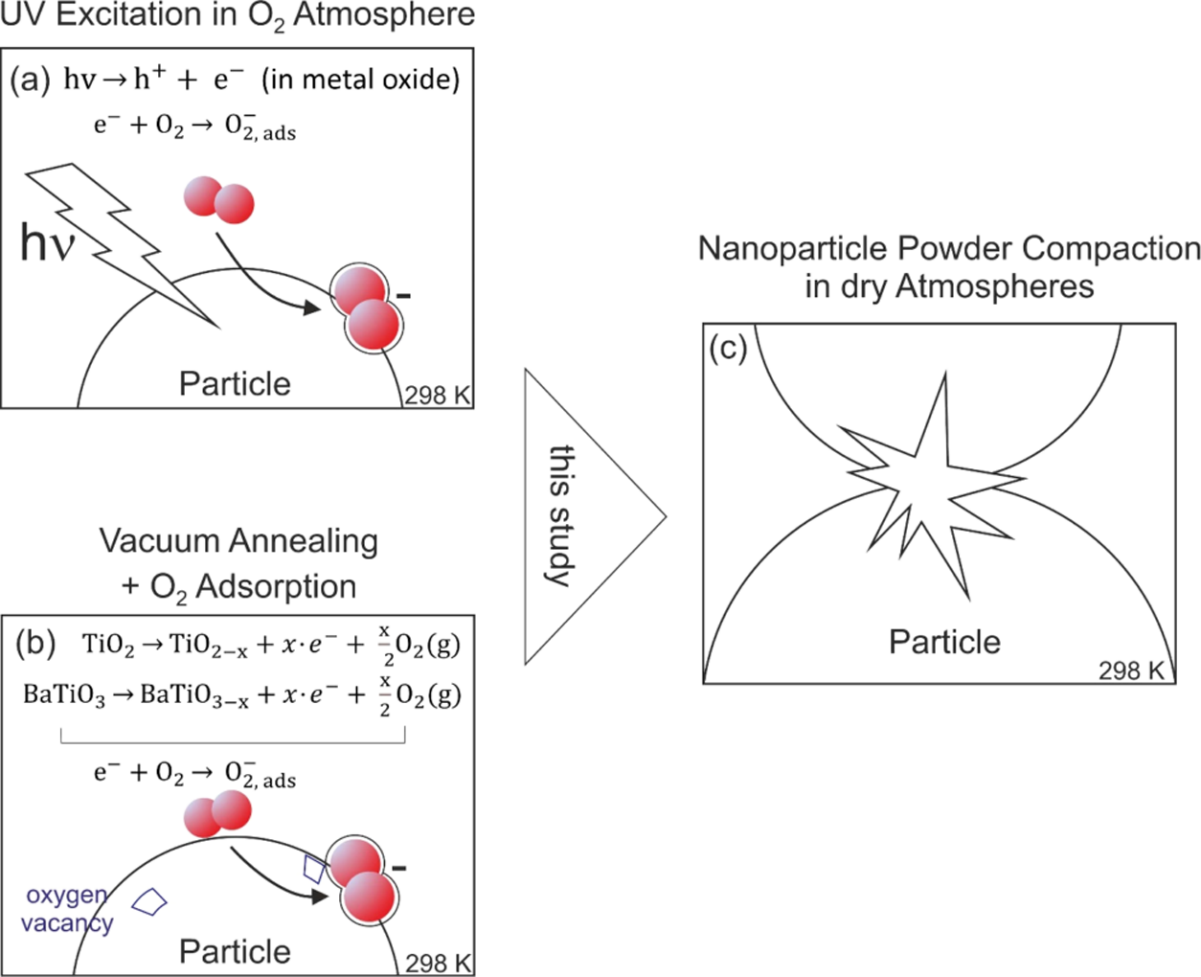
Approaches to generate superoxide anions, O_2_^–^ species, that remain adsorbed on TiO_2_ and BaTiO_3_ nanoparticles, where they serve as EPR active surface probes; (a) photoexcitation of nanoparticle powders in the presence of an O_2_ gas serving as an electron scavenger; (b) vacuum annealing of a powder to induce oxygen deficiency and, concomitantly to achieve, electronic reduction of the TiO_2_–_*x*_ or BaTiO_3–*x*_ lattice. At low deviations from stoichiometry 0 ≤ *x* ≤ 0.01, subsequent materials contact with O_2_ gas at room temperature gives rise to the formation of O_2_^–^. This study aims at the characterization of paramagnetic property changes inside metal oxide nanoparticle powders upon powder compaction in the dark (c).

**Figure 3:**
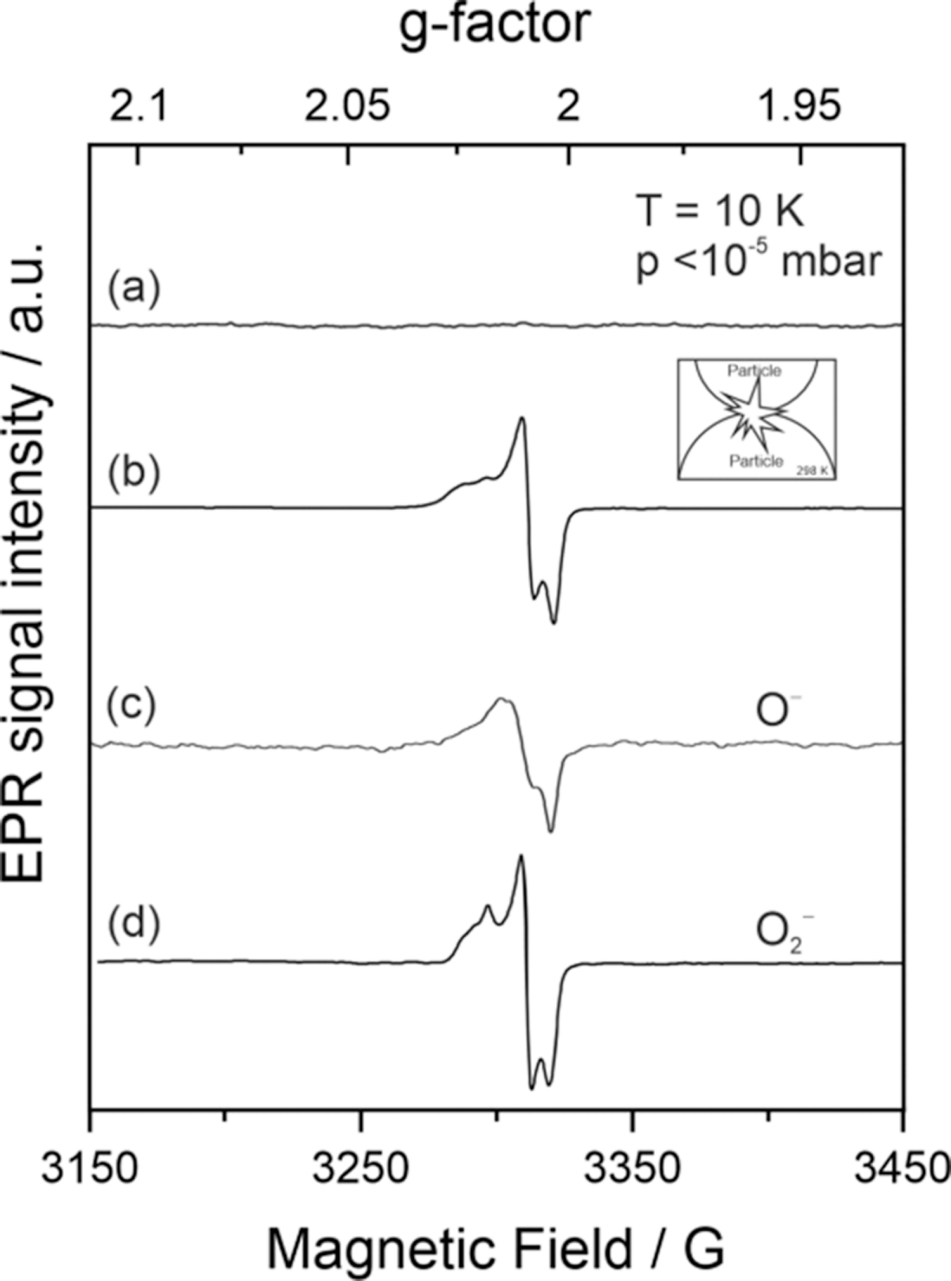
EPR spectra acquired on (a) EPR silent TiO_2_ nanoparticle powders and (b) compacts of identical powders after uniaxial powder compaction in O_2_ atmosphere, in comparison to single component spectra related to (c) trapped hole centers, O^–^ ions and (d) scavenged electrons O_2_^–^ ions (see main text for detailed explanation of their origin).

Figures 2a and b show pathways by which superoxide anions O_2_^–^ can be generated and stabilized at dehydroxylated metal oxide surfaces.^21^ On the one hand, the electron affinity of molecular oxygen is sufficient, so that through excitation from the valence into the conduction band, photogenerated electrons transfer to adsorbed oxygen. Formed in this way and provided that there are no other reaction partners such as H_2_O present,^18^ these O_2_^–^ ions are stable and can be detected with EPR spectroscopy for hours without significant concentration changes. In some metal oxides, the electron holes as the opposite type of charge carriers trap at specific defects and become accessible to EPR detection as paramagnetic O^–^ radicals. As shown below (Figure 3), corresponding resonance signals overlap with those of the O_2_^–^ ions, and spectrum simulation is typically used for analysis and determination of the symmetry and the g parameters of the individual signals.

As an alternative approach, the thermal treatment of metal oxide nanoparticle powders in atmospheres, such as vacuum, N_2_, or Ar gas, induces the depletion of lattice oxygen and generates oxygen vacancies (Figure 2b). In semiconducting metal oxides such as TiO_2_ or BaTiO_3_ and for reasons of electroneutrality, anion vacancy formation is associated with the emergence of electronically reduced Ti^3+^ (3d^1^) centers. This results from the fact that oxygen depletion from the anionic sublattice corresponds to removal of neutral oxygen species and occurs via the release and redistribution of electrons 2e^–^ (per lattice O^2–^ ion eliminated) over the surrounding lattice (Figure 2b). Previous studies on TiO_2_ and BaTiO_3_ nanoparticles with particle sizes below 20 nm (Figure 1) have shown that vacuum annealing at temperatures between 873 and 973 K and for dwell times of *t* ≤ 2 h produces only slight deviations from stoichiometry in the range 10^–5^ ≤ *x* ≤ 10^–4^.^36,37^

At room temperature and below, molecular oxygen adsorbs at the surfaces of electronically reduced metal oxide particles, accepts the electrons, which were localized at the oxygen anions prior to oxygen depletion, and reoxidizes the Ti^3+^ centers (Figure 2b). Although for room temperature experiments there is no evidence for the effective filling of anion vacancies with oxygen atoms originating from the adsorbate, the process of the formation of the O_2_^–^ can be interpreted as reoxidation of the particle surfaces. After application of this route characteristic EPR fingerprints reveal results that are very similar to those obtained after powder photoexcitation in an O_2_ atmosphere (Figure 2a).

After oxidation treatment powders of stoichiometric and impurity-free TiO_2_ nanoparticles are EPR silent and do not show any type of paramagnetic resonance in a X-band spectrum (Figure 3a, with approximately 10^16^ nanocrystals inside the EPR tube). The situation is different for pellets obtained through compaction of identical powders in an O_2_ atmosphere. For these experiments, pellets were broken, and related fragments were collected inside the volume of an EPR tube which is then connected to a high vacuum pumping rack. Continuous pumping down to a base pressure of *p* < 10^–5^ mbar enables the measurement of characteristic EPR powder spectra that point to the emergence of two distinct oxygen radicals (Figure 3b). On the basis of their g parameters (Table 1), the signal can be deconvoluted into two contributions, (i) into an axially symmetric signal that corresponds to an electron-hole center that is trapped at a surface oxygen anion (O^–^ radical, Figure 3c)^22,38^ and (ii) a spin center with rhombic local symmetry that is characterized by its g_*xx*_, g_*yy*_, and g_*zz*_ components (Table 1). The latter signal contribution is perfectly consistent with the EPR fingerprint of superoxide anions (O_2_^–^) which were introduced along Figures 2a and b and Table 1.

**Table 1:** EPR Parameters of O^–^ Ions (Trapped Hole Centers) and O_2_^–^ Ions (Scavenged Electrons) Observed on TiO_2_ Nanoparticle Surfaces

spin center	g tensor principal values			ref
	**g**_**⊥**_	**g**_**||**_		
trapped holes: O^–^ (axial symmetry)	2.012(1)	2.004(6)		ref (22)
	**g**_**xx**_	**g**_**yy**_	**g**_**zz**_	
scavenged electrons: O_2_^–^ (orthorhombic symmetry)				ref (22)
O_2_^–^ [I]	2.003(3)	2.009(6)	2.024(8)	
O_2_^–^ [II]	2.003(3)	2.009(6)	2.018(4)	

Two signals of individual spin centers are added to Figure 3 to demonstrate the individual contributions of trapped hole centers (O^–^) and scavenged electrons (O_2_^–^) in detail. As spectroscopic states, they were measured on identical nanocrystalline TiO_2_ materials, which were either exposed to UV irradiation in vacuum and at cryogenic temperatures (10 K) to generate O^–^ centers (Figure 3c) or isolated after interfacial electron transfer from vacuum-annealed (nonstoichiometric) TiO_2_ to molecular oxygen at room temperature to generate O_2_^–^ (Figures 2b and 3d).

Fragments of the nanoparticle powder compacts were subjected to subsequent UV photoexcitation at room temperature and in oxygen atmosphere for further analysis of the paramagnetic sample properties (see Figure S1 in the Supporting Information). After UV exposure for 60 min, the intensity of the composite signal that resembles that in Figure 3b) has increased by a factor of 2 without any change in the signal shape. Apparently, compaction of TiO_2_ nanoparticles leads to identical charge separation effects and paramagnetic product states as photoexcitation of the material with photon energies exceeding the band gap energy of the metal oxide (*E*_bg_ ≥ 3.2 eV). If such a sample is exposed to H_2_O vapor at room temperature (see Figure S1c in the Supporting Information)—paramagnetic surface centers typically convert into diamagnetic products in this way^18^—the signal intensity decreases down to the value measured directly after powder compaction (Figure 3b). Thus, we conclude that the additional paramagnetic centers produced by UV photoexcitation reside in the compact’s outer region where respective surface sites are accessible to both oxygen (after charge separation and during scavenging of photogenerated electrons) and water molecules from the atmosphere (during radical conversion into diamagnetic products). The inner region of the compact, where the radicals have emerged upon powder pressing, however, appears to be screened from further surface reactions and interfacial charge transfer steps.

**Figure 4:**
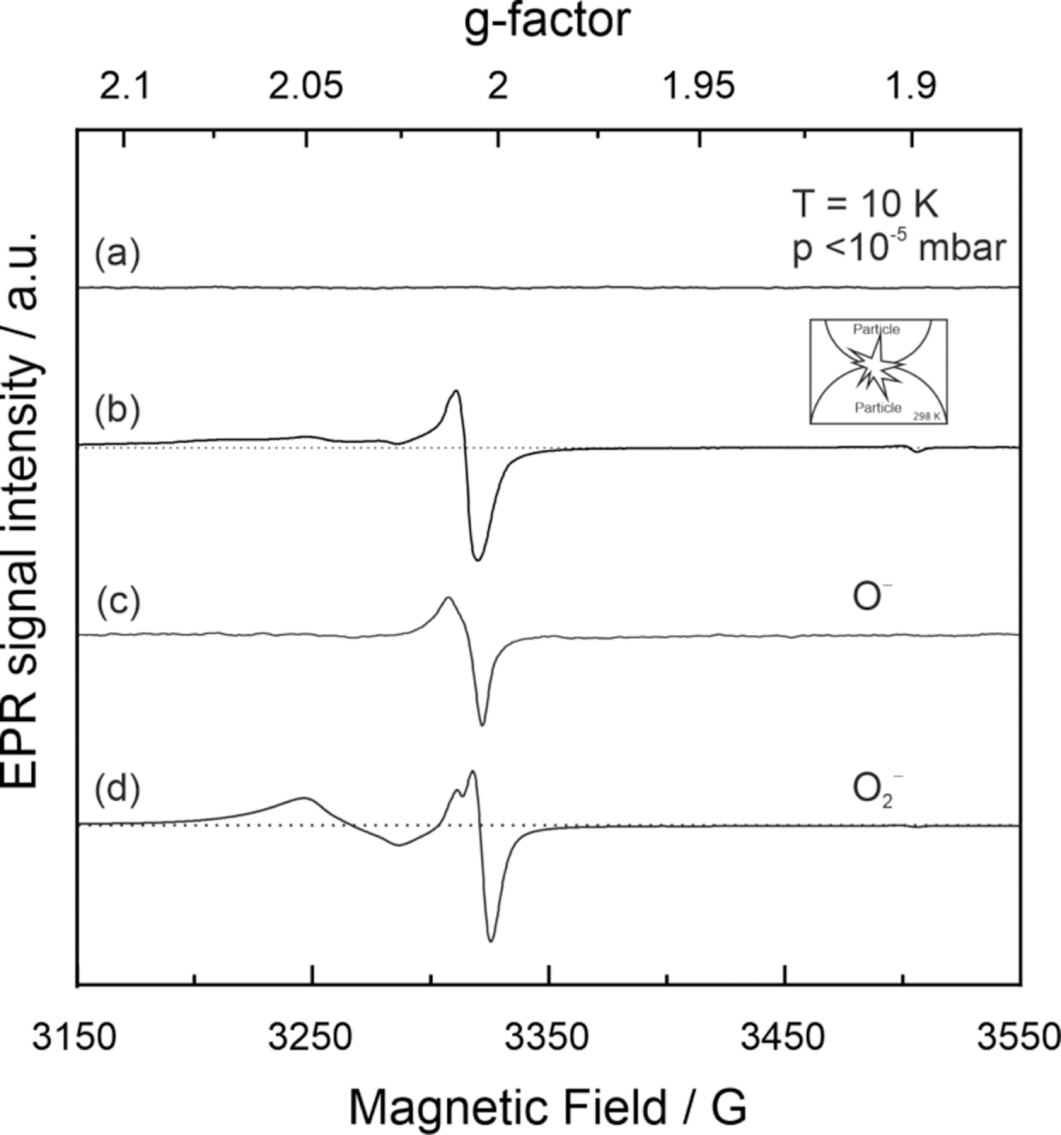
EPR spectra acquired on (a) EPR silent BaTiO_3_ nanoparticle powders and (b) compacts of identical powder after uniaxial powder compaction in an O_2_ atmosphere in comparison to single component spectra related to (c) trapped hole centers, O^–^ ions, and (d) scavenged electrons, O_2_^–^ ions.

Pristine BaTiO_3_ nanoparticle powders are exempt from paramagnetic defects and a flat line in the respective magnetic field range is measured (Figure 4a). Uniaxial pressing of nanoparticle powders, however, produces significant spectral changes (Figure 4b). The overall signal extends over a magnetic field range between 3150 and 3350 G and essentially originates from two types of paramagnetic resonance contributions (Table 2): the broad resonances below 3300 G point to the pressure-induced formation of superoxide anions O_2_^–^ (in an end-on adsorption configuration).^27^ The composite signal above 3300 G contains superimposed resonance contributions from electron–hole centers (O^–^ ions) as well as the g_*yy*_ and g_*xx*_ resonance components of a second type of superoxide anion center (O_2_^–^) that adopts a side-on adsorption configuration.^27^ Related signals were identified in previous BaTiO_3_ photoexcitation studies on similar nanoparticle powders with the emergence of electron–hole centers, on the one hand, and electrons trapped by molecular oxygen, on the other (Table 2).

**Table 2:** EPR Parameters of O^–^ Ions (Trapped Hole Centers), O_2_^–^ Ions (Scavenged Electrons), and Ti^3+^ Ions (Trapped Electrons) Observed on BaTiO_3_ Nanoparticle Surfaces and Interfaces

spin center	g tensor principal values			ref
	**g**_**⊥**_	**g**_**||**_		
O^–^ trapped hole center (axial symmetry)	2.012(6)	2.001(6)		ref (27)
	**g**_**xx**_	**g**_**yy**_	**g**_**zz**_	
Ba^2+^·O_2_^–^ superoxide anion (orthorhombic symmetry)	2.003(0)	2.008(8)	2.1(0)	ref (27)
	**g**_**⊥**_	**g**_**||**_		
O_2_^–^ superoxide anion (axial symmetry)	2.047(0)	2.026(0)		ref (27)
	**g**_**⊥**_	**g**_**||**_		
Ti^3+^ c-type polaron (axial symmetry)	1.996(8)	1.899(7)		ref (37)

The single component signals of O^–^ radicals (Figure 4c), as they can be generated by UV excitation in vacuum and at 10 K, and superoxide anions O_2_^–^, i.e., oxygen radicals, which emerge upon addition of molecular oxygen to electronically reduced barium titanate powders after previous vacuum treatment at 973 K (Figure 4d), were included in this figure. The position of the individual signal components is in very good agreement with the signals measured on the compacted sample (Figure 4b).

**Figure 5:**
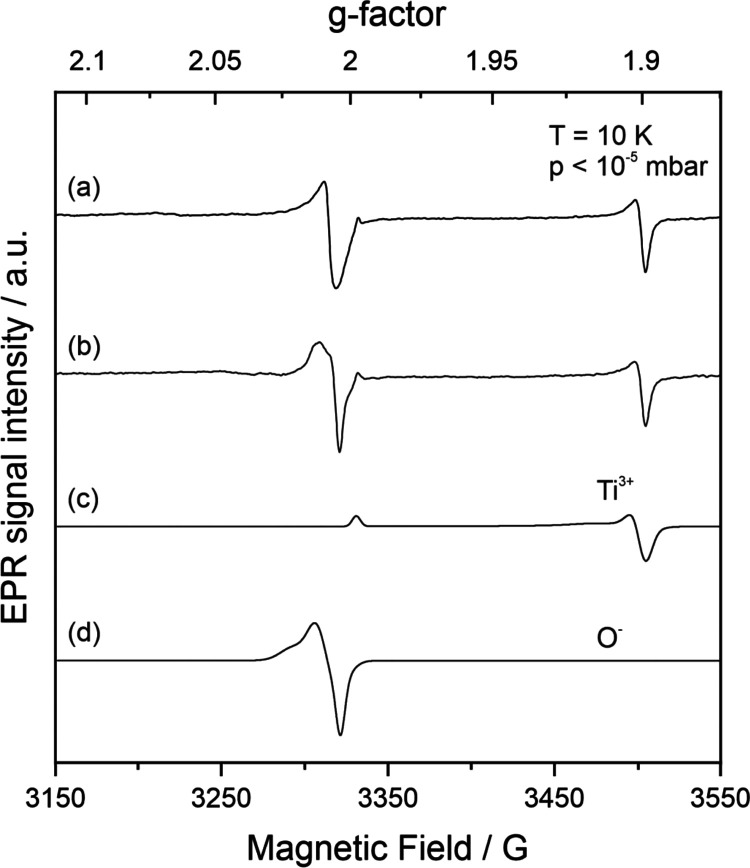
EPR spectrum of (a) BaTiO_3_ nanoparticle compacts after uniaxial powder compaction in Ar atmosphere and (b) nanoparticle powders photoexcited with UV light at 10 K. Traces (c) and (d) are the simulated single component spectra related to trapped electrons (Ti^3+^ polarons) and hole centers (O^–^ centers).^27^

Whether the compaction-induced charge separation states can be isolated in the absence of electron–hole or electron scavengers and at room temperature was addressed as a follow-up question: previous studies have shown for TiO_2_ nanoparticle powders that UV excitation at *T* = 77 K and below leads to a concentration build-up of trapped electrons (polarons) and hole centers (O^–^ centers).^38,39^ However, related charge carriers recombine and annihilate upon sample warming to room temperature.^38^ BaTiO_3_ nanoparticle powders behave differently when they are compacted into pellets under the corresponding conditions. Signals with g parameters (Table 2) that are indicative of Ti^3+^ (polarons) and O^–^ centers arise from compaction in an Ar atmosphere (Figure 5a). The underlying charge separation states remain stable under such conditions.

A spectrum with identical EPR fingerprints that are linked to Ti^3+^ (polarons) and electron–hole centers^27^ can be acquired after UV excitation of BaTiO_3_ nanoparticles with UV light at 10 K (Figure 5b-d).

Obviously, the lattice of BaTiO_3_ nanocrystals including the point defects contained therein keep the charges more effectively separated from each other than the TiO_2_ anatase lattice. Related to the materials’ relative dielectric constants, a possible explanation for this observation is the incipient ferroelectricity in the BaTiO_3_ lattice. This phenomenon, however, is naturally different in nanoparticles than in extended ferroelectric solids. Uncompensated charges located at nanocrystal surfaces can generate a depolarizing field that can be strong enough to suppress spontaneous polarization and prevent them from exhibiting a ferroelectric state. Shielding the depolarizing field and stabilizing spontaneous polarization in nanostructures with ferroelectric order is therefore a research topic of increasing scientific interest. It is worth mentioning that in ferroelectric BaTiO_3_ nanoparticles, topological polar structures have only recently been detected at the single-atom level by atomic electron tomography.^40^ Thus, there is direct experimental evidence that supports the idea that nanoparticles can sustain spontaneous polarization. The present work was carried out either in anhydrous O_2_/Ar atmospheres or under dynamic high vacuum conditions, i.e., in the absence of adsorbed dipole molecules that potentially generate a depolarizing field at the grain surfaces. The above-mentioned evidence of topological polar structures in nanoparticles is highly relevant for our qualitative understanding of the observation that the tribochemically induced charge separation effect is more pronounced and more stable, in comparison to that in TiO_2_ nanocrystals. Moreover, it is also helpful for the interpretation of the quantitative yield of separated and trapped charges, as shown in Table 3.

**Table 3:** Compaction-Induced Charge Separation Yield on MgO Nanocubes (*d* < 20 nm), TiO_2_ (*d* < 20 nm), and BaTiO_3_ (*d* < 20 nm) Nanoparticles

sample	spins per particle	source
MgO	1.5 ± 0.55	ref (17)
TiO_2_	0.2 ± 0.05	this work
BaTiO_3_	5.5 ± 2.0	this work

Including the results from previous work on MgO nanocrystals^17^ the estimated number of spins per particle was determined for TiO_2_ and BaTiO_3_ nanocrystals by EPR (second and third row in Table 3). The results in Table 3 clearly reveal that after powder compaction and tribochemical activation of BaTiO_3_ nanocrystals, they show by far the highest charge separation yield and exceed that observed for MgO by a factor of 3–4 (Table 3). Ultimately, a question related to the excitation energies required to induce the charge separation processes reported here arises. UV photoexcitation experiments of the nanoparticle powders (Figure 5b, Figures S1 and S2, Supporting Information, ref (17)) have shown that the same paramagnetic defects form in this way as with powder compaction in the dark. However, these UV excitation-induced processes require photon energies of *h*ν > 4.6 eV for MgO, to excite low-coordinated surface ions at the nanocube interfaces, or photon energies about *h*ν ≥ 3.2 eV for the electrons to pass the optical band gap of BaTiO_3_ and TiO_2_.^27^ An analysis was performed for different contact configurations between MgO nanocubes at a quantum level^17^ in order to determine the energy balance related to tribochemically induced charge separation and consecutive charge transfer. While for MgO the energy costs computed are below 4.6 eV, they still require energies ≥1.9 eV. The flexoelectric effect occurs in essentially all types of dielectrics and has been put forward as an additional driving force for tribocharge separation and transfer.^17^ Flexoelectricity corresponds to an electromechanical coupling mechanism between electric polarization and strain gradients in the dielectrics. The influence of strain gradients can become significant in nanometer-sized solids, which, in turn, leads to pronounced flexoelectric effects. BaTiO_3_ nanoparticles are very likely candidates for flexoelectric materials components. They are piezoelectric and transduce mechanical load into a flux of reactive radical species.^19^ Previous studies in the field of piezocatalysis, sonochemistry, and particle comminution have provided indirect evidence for the emergence of paramagnetic species that subsequently undergo consecutive reactions with organic monomers, water molecules, or other surface-absorbed contaminants. For the first time, this work provides direct evidence for the generation of oxygen radicals that directly result from powder compaction-induced charge separation at contact interfaces of ferro- (BaTiO_3_) or paraelectric (TiO_2_) metal oxide nanoparticles. Capturing these very early stages of an intergranular and tribochemically initiated radical chemistry experimentally opens a completely new opportunity region for the design of ferroelectric nanocomposites with flexoelectric performance. The utilization of new and emergent functional phenomena such as flexo-resistance or the combined flexo- and photoelectric material performance of catalysts and electronic devices will benefit from such nanoparticle-derived insights.

## Conclusions

Local mechanical stress at the asperities of ferroelectric BaTiO_3_ and paraelectric TiO_2_ nanoparticle surfaces in contact that arises from powder compaction generates paramagnetic defects as evidence for tribochemically induced charge separation. When compacted in the presence of oxygen (O_2_) as an electron scavenger, paramagnetic oxygen radicals—specifically trapped hole centers (O^–^) and scavenged electrons (O_2_^–^)—can be detected on the nanoparticle surfaces using EPR spectroscopy. The comparison of ferroelectric BaTiO_3_ with paraelectric TiO_2_ nanoparticles shows that spontaneous polarization effects of the BaTiO_3_ lattice significantly increase the yield of radicals by a factor of more than 20 (Table 3). The study also reveals that high vacuum treatment at elevated temperatures is effective to eliminate water and other adsorbates from the particle surface to such an extent that intrinsic defects, such as electron–hole centers, can be isolated and detected at the particle surfaces. This creates a unique starting point for the stepwise investigation of the reactivity of tribochemically activated surface sites toward monomers and other species that can undergo consecutive radical reactions.

## Supporting Information Available

EPR spectra of TiO_2_ nanoparticle compacts after compaction in O_2_; UV excitation in O_2_; and after subsequent exposure to water vapor (PDF)
