# Supplementary material for: BaTiO3 Nanoparticle Interfaces in Contact: Ferroelectricity Drives Tribochemically Induced Oxygen Radical Formation
Source: Langmuir. 2024 Dec 13;40(51):26928–35. doi: 10.1021/acs.langmuir.4c03390 (PMC11673569; doi:10.1021/acs.langmuir.4c03390)
Supplement: Supplementary file 1 — la4c03390_si_001.pdf [file la4c03390_si_001.pdf]

Supporting Information

$BaTiO_3$ Nanoparticle Interfaces in Contact: Ferroelectricity Drives Tribochemically induced Oxygen Radical Formation

Korbinian Aicher[a], *Thomas Berger*[a], *and Oliver Diwald*[a,*]

[a] Department of Chemistry and Physics of Materials,

Paris-Lodron University Salzburg, Jakob-Haringer-Straße 2a,

A-5020 Salzburg, Austria

E-mail: oliver.diwald@plus.ac.at

Keywords: mechanochemistry, charge separation, rubbing powders, tribochemistry, defect engineering;

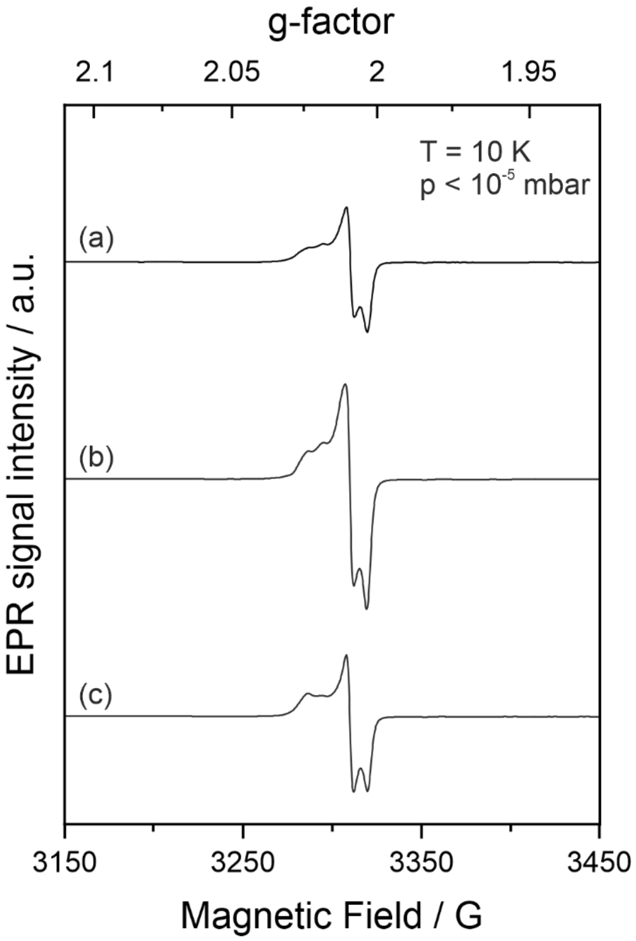

Figure S1: EPR spectra acquired on (a) TiO_2 nanoparticle compacts after uniaxial powder compaction in O_2 atmosphere, (b) after 60 min UV light in 30 mbar O_2 at room temperature and (c) after subsequent sample exposure to 30 mbar H_2O (g) at room temperature for an exposure time of 40 minutes.

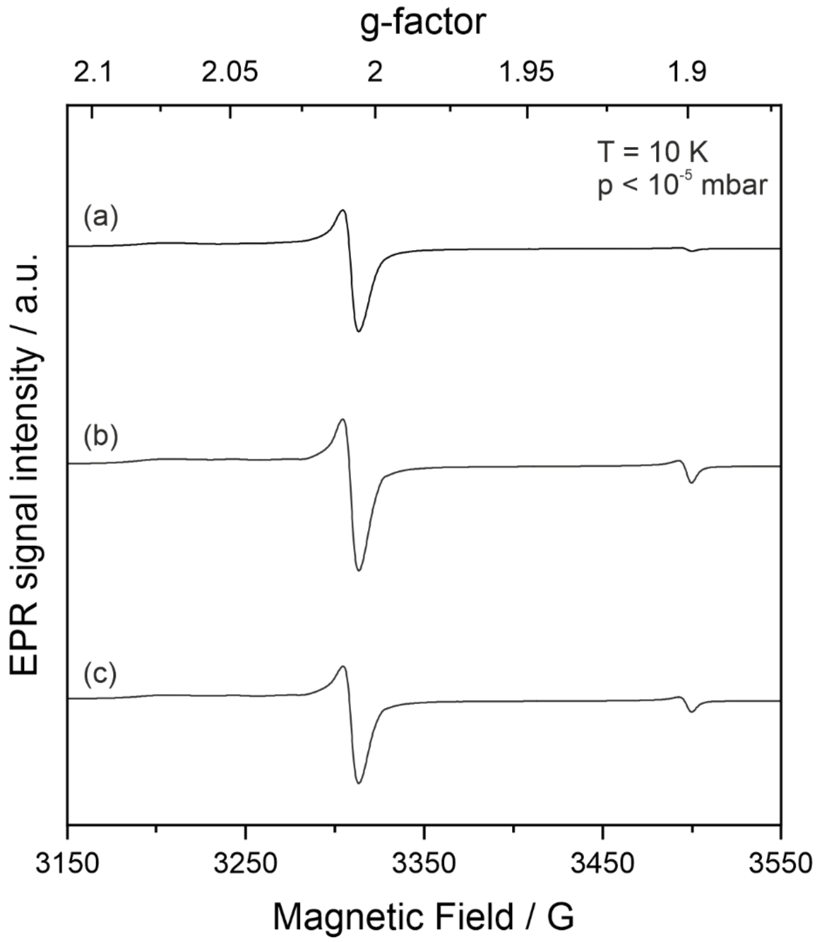

Figure S2: EPR spectra acquired on (a) $BaTiO_3$ nanoparticle compacts after uniaxial powder compaction in O_2 atmosphere, (b) after 60 min UV light in 30 mbar O_2 at room temperature and (c) after subsequent sample exposure to 30 mbar H_2O(g) at room temperature for an exposure time of 40 minutes.
